# A phase 2 study of vorinostat in locally advanced, recurrent, or metastatic adenoid cystic carcinoma

**DOI:** 10.18632/oncotarget.16464

**Published:** 2017-03-22

**Authors:** Priscila H. Goncalves, Lance K. Heilbrun, Michael T. Barrett, Shivaani Kummar, Aaron R. Hansen, Lillian L. Siu, Richard L. Piekarz, Ammar W. Sukari, Joseph Chao, Mary Jo Pilat, Daryn W. Smith, Lindsay Casetta, Scott A. Boerner, Alice Chen, Elizabeth Lenkiewicz, Smriti Malasi, Patricia M. LoRusso

**Affiliations:** ^1^ Karmanos Cancer Institute, Wayne State University, Detroit, MI, USA; ^2^ Mayo Clinic Arizona, Scottsdale, AZ, USA; ^3^ Division of Medical Oncology and Hematology, Princess Margaret Cancer Centre, University of Toronto, Toronto, ON, Canada; ^4^ Division of Cancer Treatment and Diagnosis, National Cancer Institute, Bethesda, MD, USA; ^5^ Department of Medical Oncology and Therapeutics Research, City of Hope Comprehensive Cancer Center, Duarte, CA, USA; ^6^ Eugene Applebaum College of Pharmacy and Health Sciences, Physician Assistant Studies, Wayne State University, Detroit, MI, USA; ^7^ Current address: Yale Cancer Center, New Haven, CT, USA; ^8^ Current address: Stanford University, Palo Alto, CA, USA

**Keywords:** adenoid cystic, salivary gland tumor, vorinostat, suberoylanilide hydroxamic acid, SAHA

## Abstract

**Purpose:**

Vorinostat is a histone deacetylase inhibitor (HDACi). Based on a confirmed partial response (PR) in an adenoid cystic carcinoma (ACC) patient treated with vorinostat in a prior phase 1 trial, we initiated this phase 2 trial. Methods: Vorinostat was administered orally 400 mg daily, 28 day cycles. The primary objective was to evaluate response rate (RR). Exploratory studies included whole exome sequencing (WES) of selected patients.

**Results:**

Thirty patients were enrolled. Median age of patients was 53 years (range 21–73). Median number of cycles was 5 (range 1-66). Lymphopenia (*n* = 5), hypertension (*n* = 3), oral pain (*n* = 2), thromboembolic events (*n* = 2) and fatigue (*n* = 2) were the only grade 3 adverse events (AEs) that occurred in more than 1 patient. Eleven patients were dose reduced secondary to drug-related AEs. Two patients had a partial response (PR), with response durations of 53 and 7.2 months. One patient had a minor response with a decrease in ascites (for 19 cycles). Stable disease was the best response in 27 patients. Targeted and WES of 8 patients in this trial identified mutations in chromatin remodeling genes highlighting the role of the epigenome in ACC. Conclusion: Vorinostat demonstrated efficacy in patients with ACC supporting the inclusion of HDACi in future studies to treat ACC.

## INTRODUCTION

Adenoid cystic carcinoma (ACC) is a rare type of cancer, most commonly originating from the salivary glands, with an indolent behavior but a high propensity to metastasize [1]. Surgery with wide resection is the mainstay of treatment for localized ACC. A watch and wait approach is appropriate if a patient has metastases, especially if confined to the lungs with minimal symptoms [2].

Several anti-cancer agents have been studied to treat metastatic ACC, however none have shown a robust response rate, with stable disease (SD) being the most common reported outcome. SD duration over 6 months is a common metric used in ACC trials, varying among different agents, with reported ranges of 30–60% (reviewed in [3]). Currently, there is no Food and Drug Administration (FDA) approved agent for the treatment of ACC.

Suberoylanilide hydroxamic acid (SAHA), also known as vorinostat, is a small molecule inhibitor of histone deacetylase (HDAC). Two patients with ACC on a previously published National Cancer Institute (NCI) trial evaluating vorinostat in patients with advanced cancers and liver dysfunction experienced significant radiological and/or clinical improvement [4]. Additionally, recent next generation sequencing (NGS) studies of ACC have demonstrated a low mutation rate with few recurring single gene mutations converging on regulators of chromatin remodeling [5, 6].

Based on these encouraging findings, we undertook this multi-institutional phase 2 trial of vorinostat in patients with locally advanced or metastatic ACC.

## RESULTS

### Baseline demographics and patient characteristics

Thirty patients were enrolled over 22 months. As shown in Table 1, 19 patients (63%) were female and 24 (80%) were Caucasian. The median age was 53 years (range 21–73), 21 had a performance status of 1 and 19 were chemo-naïve. Twenty-eight had metastatic disease and 2 patients had locally advanced disease. While not an eligibility criterion, 27 out of 30 patients had radiographic disease progression before study entry.

**Table 1:** Baseline demographics and disease characteristics (N = 30)

Characteristics	No. of Patients	%
**Race**		
White	24	80
African-American	3	10
Asian	2	6.7
Middle Eastern	1	3.3
**Sex**		
Male	11	37
Female	19	63
**ECOG PS**		
0	7	23
1	21	70
2	2	7
**Age (years)**		
Median (range)	53 (21–73)	
**Disease Site**		
Metastatic	28	93
Locally Advanced	2	7
**Prior Chemotherapy**		
Chemo-naive	19	63

ECOG PS- Eastern Cooperative Oncology Group performance status

### Efficacy

**Figure 1:**
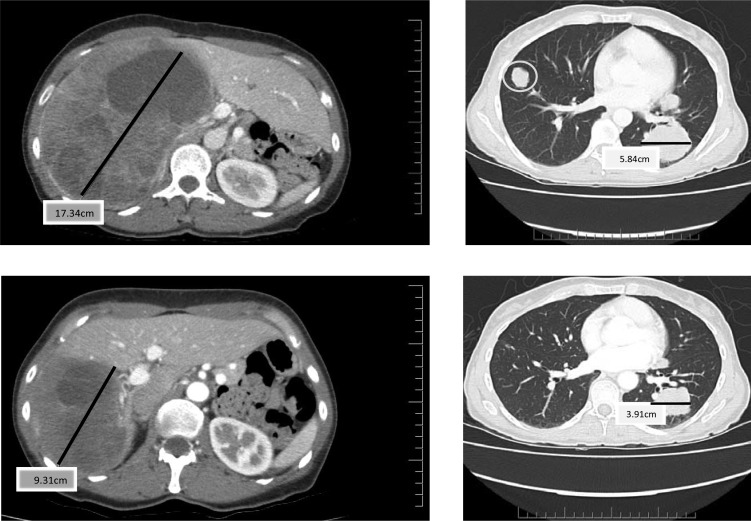
Baseline (top) and Cycle 13 (bottom) computer tomography (CT) scan (chest and abdomen) from a patient who had a partial response and a duration of 7.2 months

**Figure 2:**
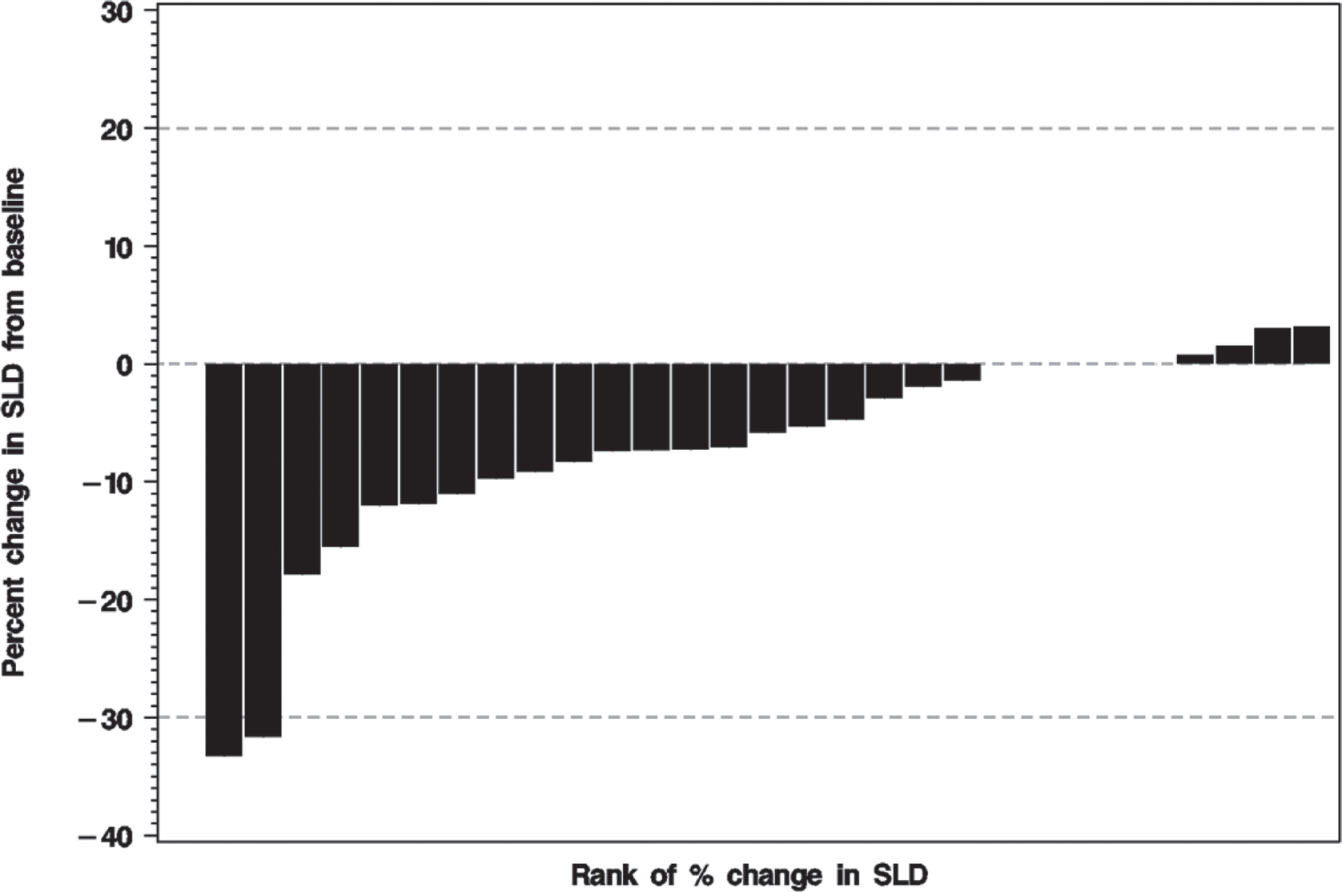
Waterfall plot of the percent change from baseline to best response in the sum of the longest diameters (SLD) of the index lesions for 29 treated patients For one treated patient the follow-up CT was not available. The horizontal dashed lines indicate the thresholds for partial response (PR: ≥ 30% decrease in SLD) and for progressive disease (PD: ≥ 20% increase in SLD).

Partial responses by Response Evaluation Criteria in Solid Tumors (RECIST) were observed in two patients. These responses were not immediate. One patient had a decrease in the size of lung nodules achieving a partial response (PR) at cycle 8 and the other patient experienced reduction in size and number of liver and lung lesions achieving a PR by cycle 10 (Figure 1). One of the patients with PR had surgery and radiation as prior therapies and the other patient with PR also received surgery, radiation and one systemic chemotherapy prior to enrolling in this trial. While stable disease (SD) was the best response in 27 patients, 20 patients demonstrated a decrease in the size of their tumors (Figure 2). Another patient had improvement in ascites from cycle 6 through cycle 20 (minor response). The overall response rate (RR) was 7% with a clinical benefit rate (PR+SD) of 97% (Table 2). Anecdotal improvement in symptoms was also noted in 3 patients despite SD by RECIST. Three patients received extensive therapy to 56, 62 and 66 cycles.

**Table 2:** Response rate (among N = 30 treated patients)

Response Category	No. of Patients (%)	95% Confidence Interval
**Partial Response (PR)**	2/30 (7%)	2%–21%
**Stable Disease (SD)**	27/30 (90%)	74%–97%
**Clinical Benefit (PR+SD)**	29/30 (97%)	83%–99%
**Progressive Disease**	1/30 (4%)	1%–17%

### Toxicity

The most frequent grade 3 toxicities were lymphopenia (7/30 = 23%), hypertension and fatigue (3/30 = 10% each), headache, oral pain and thromboembolic event (2/30 = 7% each) (Table 3). Grade 4 bronchopulmonary hemorrhage and hypoxia occurred in 3% (1 patient each), but neither was drug-related. Eleven patients were dose reduced, 10 patients were reduced by one dose level to 300mg daily and 1 patient to 300 mg once a day for 5 days with 2 days off (reduction of two dose levels). Three patients discontinued treatment due to toxicity.

**Table 3:** Grade 3 or 4 toxicities experienced by at least 1 patient (N = 30 treated patients)

Type of toxicity ^a^	Worst grade experienced				
	0	1	2	3	4
Bronchopulmonary hemorrhage	0	0	0	0	1
Hypoxia	0	0	0	0	1
Lymphopenia	13	7	3	7	0
Hypertension	15	5	7	3	0
Fatigue	9	12	6	3	0
Headache	21	4	3	2	0
Oral pain	27	0	1	2	0
Thrombo-embolic event	26	2	0	2	0

^a^There were 14 other miscellaneous toxicities whose worst Grade was 3, and they occurred in only 1 patient: appendicitis, menorrhagia, atelectasis, bronchial obstruction, pneumonia, lung infection, wound complication, thrombocytopenia, diarrhea, cataract, oral mucositis, hypophosphatemia, increased lymphocyte count, and non-cardiac chest pain.

### Time to event end-points

Time to tumor response (TTR) was 7.7 and 10.0 months in the 2 patients who achieved PR. The duration of their response was 53 and 7.2 months, respectively, with the former patient still receiving study drug. Prior to study entry, 27 (90%) of the 30 patients had documented progressive disease on their most recent scans. The median follow-up among the censored patients was 8.0 months for stable disease duration (SDD), 10.0 months for progression free survival (PFS), and 11.5 months for overall survival (OS). The 6 month rates for SDD, PFS, and OS were 75%, 72%, and 94%, respectively. The 12 month rates for SDD, PFS and OS were 45%, 46%, and 88%, respectively. (Supplementary Table 1 - Supplementary Appendix). The median PFS and SDD were both 11.4 months, and the median OS has not been reached. The estimated SDD and PFS distributions are shown in the Supplementary Appendix (Supplementary Figures 1 and 2, respectively).

### Exploratory studies

We hypothesized that the variation in clinical response to vorinostat (“responders” or “non-responders”- Table 4) is associated with the presence or absence of driver mutations including those targeting chromatin remodeling genes involved in the regulation of the cancer epigenome. To test our hypothesis, we used both targeted and whole exome sequencing to survey the mutational landscape of each tumor.

**Table 4:** Selected responses to vorinostat and mutations identified

	001-002^a,b^	001-004^a,b^	001-006^a,b^	003-014^a,b^	003-015^b^	003-018^b^	004-023^b^	003-027^a,b^	
Clinical Response/Number of Cycles Received	Decrease in neurological pain - 44 cycles	Partial response by RECIST- 66 cycles	Decrease in ascites and abdominal girth −19 cycles	Prolonged stable disease – 57 cycles	Prolonged stable disease −51 cycles	Progressed on study after 2 cycles	Prolonged stable disease −22 cycles	Progressed on study after 3 cycles	
ARID3A									Chromatin remodeling
ARID4B									
BRD1									
BRD3									
KDM4D									
KDM6A									
KMT2A									
KMT2E									
FAM129B									
PHF1									
PHF2									
PRDM1									
PRDM16									
SMARCA2									

CDK8									Cell signaling
DLL1									
ESR1									
FAT3									
FZD10									
JAG1									
MAP3K1									
MTOR									
NOTCH1									
OGT									
PIK3CA									
RASA3									
RHBDF1									
SIK3									

CDC25C									Cell cycle DNA damage
ERCC2									
FANCC									
MLF1									
N4BP2									
PLK4									
POLE									
RAD52									
SH2D4A									

RECIST- Response Evaluation Criteria in Solid Tumors; Yellow- Nonsense; Red- Missense; Blue- Frame shift indel; Green- Essential splice, ^a^- Exome, ^b^- 10-gene panel.

### Exploratory studies results

#### “Responders”

We sequenced the exomes from 4 of these patients (001–002, 001–004, 001–006, 003–014). In each case we found somatic mutations of interest after filtering. These and 2 additional responder cases (003–015, 004–023) were screened with our 10 gene panel. A striking observation was the lack of recurring gene specific mutations in these 4 cases (Table 4). However, recurring cell pathways and processes were targeted.

#### Patient 001–002

There were a series of unique somatic variants detected in this tumor. Notably these converged on chromatin remodeling, transcriptional regulation, and cell signaling. The DNA binding factor *ARID3A* has been implicated in RB1/E2F mediated control of cell cycle progression while *KMT2E* and *KMT2A* belong to the myeloid/lymphoid or mixed-lineage leukemia (MLL) class of epigenetic writers (Supplementary Figure 3 - Supplementary Appendix). In addition, mutations targeted regulators of RAS (*RASA3*), EGFR (*RHBDF1*), and estrogen (*ESR1*) and NOTCH (*DLL1*) signaling.

#### Patient 001–004

Variants in genes associated with chromatin remodeling and DNA repair were detected. These included *BRCA1*^Q356R^, *BRCA2*^N372H^, and *EZH2*^D185H^. However, each of these variants appears in dbSNP, has a population frequency of > 5%, and has not been associated with an increased cancer risk [7]. We also detected *ATM*^R720H^ in the tumor tissue. However, the targeted amino acids in both wild type and this somatic variant have positive charge side chains. Thus, the functional effects of this variant on ATM activity are not clear. In contrast the *MLF1*^L120^* and *N4BP2*^K76^* non sense variants have low or absent reported allele frequencies. *MLF1* (Myeloid leukemia factor 1) is a transcription factor that prevents cells from exiting the cell cycle through suppression of CDKN1B/p27Kip1 levels and activation of TP53 [8]. Mutations in *TP53* have been reported in ACC [6]. However, we did not observe any *TP53* mutations in the current study. Thus the *MLF1*^L120^* mutation may provide a novel mechanism to target *TP53* mediated repair and cell cycle checkpoint functions in ACC. *N4BP2* (NEDD4 binding protein 2) has 5′-polynucleotide kinase and nicking endonuclease activity and may play a role in DNA repair or recombination [9].

#### Patient 001–006

We identified two additional novel ACC mutations (*SH2D4A*^R237^*, *FZD10*^G345^*) that have previously been reported in colon and gastric cancer [10–13]. *SH2D4A* inhibits estrogen-induced cell proliferation by competing with phospholipase C, gamma 2 (PLCG) for binding to ESR1, blocking the effect of estrogen on PLCG and repressing estrogen-induced proliferation [12]. It may play a role in T-cell development and function. Both of these unique *SH2D4A* and *FZD10* mutations were validated in our targeted resequencing analyses. The Drosophila frizzled polarity gene homolog 10 (*FZD10*) is a member of the G protein coupled receptor (GPCR) superfamily, exhibiting characteristics of a WNT receptor [13]. We also detected variants in chromatin remodeler genes including *ARID4B*^T84R^ and *PHF1*^E128Q^. The latter gene encodes a Polycomb group protein that is a component of a histone H3 lysine-27 (H3K27)-specific methyltransferase complex, and functions in transcriptional repression of homeotic genes [14]. The protein is also recruited to double-strand breaks, and reduced protein levels results in X-ray sensitivity and increased homologous recombination [15, 16]. Recurring chromosomal aberrations involving *PHF1* may be a cause of endometrial stromal tumors [17, 18]. In addition, we detected variants in the DNA damage checkpoint regulators *ATR*^Phe926Leu^ and *RAD52*^Y415^*.

#### Patient 003–014

The mutations in this tumor included 3 predicted high impact mutations in *MTOR* a regulator of stress response, *OGT* a glycosyltransferase that modifies a broad range of targets including H2B, AKT1, EZH2, PFKL, KMT2E/MLL5, MAPT/TAU and HCFC1, and *FAM129B* a negative regulator of apoptosis [19]. Additional mutations included the cell cycle regulator *CDC25C*, the chromatin regulators *PHF2* and *BRD1*, and the NOTCH1 ligand *JAG1*. The latter is a transcriptional target of MYB [20].

#### Patients 003–015 and 004–023

Both patients were screened with our 10 gene panel. We detected a somatic variant in *SMARCA2* in 003–015 adding to the list of unique mutations in chromatin regulators. In addition, we detected a *PIK3CA* mutation in 004–023. Mutations in both these genes have been previously reported in ACC [5, 6].

#### “Non-responders”

We obtained WES data from one of four non-responders (003–027). Targeted resequencing provided additional data for a second non- responder (003–018) and validation of the KDM6A mutation detected in the whole exome results (Table 4).

#### Patient 003–027

A striking finding was the presence of a mutation in the *NOTCH1* receptor (Supplementary Figure 3). This gain of function mutation destabilizes the heterodimerization domain of the receptor. It results in ligand-independent cleavage of Notch1 at site S2 and subsequent receptor activation. This mutation is a recurrent driver of T cell ALL [21, 22]. To our knowledge, this is the first report of the association of this recurring mutation in ACC with resistance to vorinostat. We also detected a nonsense mutation in the lysine demethylase *KDM6A*. Mutations in this lysine demethylase, also known as *UTX*, have been reported in several cancers and are one of several classes of mutations that are believed to converge on chromatin remodeling in ACC [11, 23]. Pathogenic KDM6A variants disrupt histone structure [23]. This latter mutation was unique to non-responder 003–027 and was validated by targeted resequencing with our 10-gene panel.

#### Patient 003–018

The exome data for the tumor sample failed overall quality metrics including coverage and percent target bases. Targeted resequencing was of sufficient quality for this tumor normal pair however we did not identify any somatic variants in the 10-gene panel.

## DISCUSSION

ACC is a rare neoplasm with an initial indolent pace. However, once it becomes metastatic, it runs an inexorable, albeit slow growing course with poor and short-lived responses to available treatments [2, 24].

In our study we saw 2 PRs by RECIST out of 30 patients treated with vorinostat. Both responses occurred late into treatment after 8 and 10 cycles. A third patient had a minor response with reduction in ascites, and eliminated the need for therapeutic paracentesis. Anedoctal improvement in symptoms was also observed in an additional 3 patients (decreased pain, improvement in shortness of breath, improvement in eye movement, and improvement in fatigue) with SD by RECIST. Of note, 20 patients demonstrated a decrease in the size of their tumors. Additionally, 2 patients with ACC were treated with vorinostat in a prior NCI liver dysfunction trial and experienced significant clinical and radiological improvement. (decrease in size of liver lesions and normalization of liver enzymes from moderate liver dysfunction) [4]. In the patients from the liver dysfunction trial, the clinical improvement (less fatigue and pain, weight gain) also started early (after 2–3 cycles) and preceded any radiologic change by several cycles, similar to what we observed in our current trial [4].

SD duration (SDD) has been described in several trials for patients with ACC [25–31]. It is unclear if SD represents a marker of drug activity or simply the indolent behavior of this tumor. However, most ACC studies report SDD of 6 months or more as an endpoint, which may be clinically relevant, especially in view of tumors that were progressing prior to study entry (reviewed in [32]). In our study, although not required as part of eligibility criteria; 90% (27/30) of the patients had radiological evidence of disease progression prior to enrollment. We observed a 6-month rate of SD of 75%, which is comparable to recent systemic therapeutic interventions studied in this disease [27–29, 33].

Currently, response to treatment in most solid tumors is evaluated by measuring the sum of the longest diameter of target lesions according to the RECIST criteria, which has several limitations [34]. RECIST does not take into account tumor growth dynamics or rate and cannot fully capture response and progression in certain tumor types such as gastrointestinal stromal tumors (GISTs), where which incorporates tumor densities in assessing responses, have been more widely used have been more widely used [35]. Additionally, in GIST, RECIST underestimates PR rates when compared to CHOI criteria [36]. A recent study of sorafenib in patients with salivary gland tumors (both ACC and non-ACC) used both RECIST and CHOI criteria to assess responses. It revealed 6 PRs according to RECIST, and 10 PRs according to CHOI (only 2 were concordant with RECIST) [33]. Also, RECIST may not be the best tool to evaluate tumor response to targeted agents or immune therapy [37–40]. The concept of volumetric tumor growth likely is a better method to assess response in such instances [41]. Volumetric imaging analysis can predict clinical response earlier than RECIST in some cancers and it is also more sensitive than changes in unidimensional diameters [41]. Considering the late responses observed in some of the patients in the current study, along with early symptomatic improvement, a possible explanation could be that RECIST criteria may not be the best method to monitor ACC patients for response.

Patient reported outcomes (PRO) questionnaires evaluate the impact on patient's functioning and well-being that could likely be caused by the disease and/or its treatment without modification or interpretation by the observer [42]. Given the slow growing but unrelenting progression of ACC, improvement in PRO is a reasonable measure of clinical benefit. Future trials in patients with ACC should incorporate such questionnaires as endpoints.

Another possibility is that the late responses we observed could be due to an immuno-stimulatory effect of vorinostat, which may take longer to occur and not be evident radiographically as early as with some other therapeutic interventions. Mounting evidence suggests a role for HDACi in modulating the immune system and enhancing efficacy of immunotherapeutic strategies. HDACi have been shown to alter the activation and function of macrophage and dendritic cells [43]; regulate cytokine production [44, 45] and upregulate major histocompatibility class I and II molecules [44, 46].

A feature of the ACC mutational landscape is a low mutational burden with a paucity of common recurring driver mutations typically seen in solid tumors [5, 6]. For example, *TP53* mutations, one of the most frequent somatic lesions in solid tumors, have been detected in only 5% of ACC cases studied [6]. Nevertheless, this represents the second highest incidence of reported somatic variants in ACC. Notably none were detected by either WES or targeted resequencing in the present study. Recent studies suggest that the disparate low frequency mutations in ACC tumors appear to converge on specific pathways notably DNA repair, chromatin regulation, and NOTCH signaling. The exception to this inter-tumor genomic diversity is a translocation targeting c-Myb and NFIB, creating a fusion gene in > 40% of ACC tumors [47]. Except for low impact variants in UTRs we did not detect any evidence for alterations (mutations or copy number aberrant intervals) associated with this frequent event in our ACC cohort. However, our experimental approach, whole exome sequences for 5 tumors and targeted resequencing that included 3 additional samples, was designed for problematic formalin-fixed, paraffin-embedded (FFPE) samples and was not optimal for detection of genomic lesions associated with translocations.

The whole exome data for the patients in this study highlight the role of DNA repair and chromatin structure regulation in ACC. Mutations, including somatic non sense mutations, targeting key mediators of repair, including RAD52, ATR, and POLQ, were detected in multiple patients. Although none of these mutations were recurring, they converge on well-characterized steps in DNA repair and replication. In addition, we detected mutations targeting different cell signaling pathways including estrogen, NOTCH and the Wnt pathway, as well as PIK3CA/AKT signaling. In each case, unique mutations converged on the pathway. These observations are in agreement with recent whole exome data of ACC [5, 6]. In addition, the validated mutation in *FZD10* provides further evidence for a role of aberrant Wnt signaling in ACC [48]. The most prominent set of mutations was present in multiple chromatin regulating genes (Table 4). These included lysine methyltransferases, bromodomain containing proteins, and members of the SWI/SNF chromatin regulator family. Variants in *SMARCA2* were detected in patient 003–015 however they were either conserved or resulted in an amino acid substitution with the same polarity.

The presence of the *KDM6A* non sense mutation in patient 003–027 is consistent with studies suggesting that disruption of the epigenome is a driver in a subset of ACC [49, 50]. Mutations that arise in this region of the protein have been described previously in multiple tumor types. The functional impact of these *KDM6A* mutations was evaluated via a well-characterized assay for trimethylation at lysine 27 of histone H3 (H3K27me3). Notably abrogation of demethylase activity was observed in cells overexpressing mutant *KDM6A* but not in those overexpressing wild-type *KDM6A*. Moreover, whereas wild-type *KDM6A* suppressed growth, mutants either lost the ability to suppress growth or, in some cases, augmented it (dominant phenotype). Strikingly patient 003–027, whose tumor had a *KDM6A*^R1272X^ high impact mutation, progressed after only 3 cycles of vorinostat. Future clinical trials should incorporate sequencing of this and other histone demethylases as a correlative for responses to vorinostat and other epigenetic targeting agents. The presence of well-characterized activating mutations in NOTCH1 supports the role of this pathway in ACC and its potential therapeutic targeting. Notably, mutations targeting NOTCH signaling were present in both responders and non-responders (Supplementary Figure 3, Table 4). Our study adds to the list of mutations reported in ACC and provides further support for the role of mutations targeting chromatin-remodeling genes in this disease.

Our small exploratory study confirms the presence of mutations targeting epigenome regulation in ACC, as 10 out of 11 patients had mutations in chromatin remodeling genes. Notably, a well characterized mutation in the histone demethylase *KDM6A/UTX* was present in a patient who did not respond to vorinostat. This is in contrast to the presence of mutations in histone methyltransferases (KMT2E and KMT2A), bromodomain containing proteins (BRD1 and BRD3), and members of the SWI/SNF chromatin regulator family (SMARCA2) in responders. However, this non-responder patient (003–027) had an activating *NOTCH1* mutation, suggesting that it could be the driving mutation in this case, as activating NOTCH1 mutations have been shown to confer a worse prognosis [51]. The presence of a well-characterized activating mutation in NOTCH1 confirms the role of this signaling pathway in ACC and may provide a therapeutic window in future studies. To advance these observations will require a more standardized tissue collection and processing protocol for correlative studies. Ongoing improvements in NGS technologies will make it feasible to recover more data from currently limited samples. In addition, the data from our current study and recently published NGS data provide the basis for well-designed gene panels that should include *KDM6A/UTX* and related histone demethylases for targeted resequencing

We acknowledge the limitations of our study. First, while not all patients had documented progression of disease at study entry, nonetheless, almost all (90%) of the patients did. Second, we did not have a quality of life questionnaire in this trial, which would have helped to capture and describe the clinical improvements observed. Third, we did not have funding to incorporate other imaging modalities, (such as PET/CT), or to utilize other measurement criteria (such as volumetric assessment), which would have helped evaluate the utility of a different imaging criteria in ACC. Fourth, due to the small number of samples analyzed, we could not unequivocally identify mutations that were predictors of response or resistance to vorinostat.

In conclusion, the findings from this study are encouraging. Vorinostat was relatively well tolerated and patients remained on drug treatment for significant periods of time. The clinical benefit rate (97%) was very high, as was the 6-month stable disease rate (75%). Despite a small number of PRs overall, these responses were durable and associated with improvement in symptoms. We found mutations in chromatin remodeling genes in most samples analyzed. Perhaps the fact that ACC has a low rate of somatic mutations, coupled with the pleotropic effects of vorinostat as a modulator of epigenetics, can partly explain this drug's antitumor activity in this disease. In the future, it will be important to assess responses to vorinostat and other HDACi in patients with ACC relative to aberrant epigenetic regulation with tumor gene sequencing. In summary, despite this being a statistically negative trial, we observed clinical improvement in symptoms in several patients, along with two partial responses and a significant decrease in ascites in another patient. We find this study encouraging and will pursue further prospective clinical trials evaluating HDAC inhibitors perhaps in combination with other agents, for the treatment of ACC we find this study encouraging and will pursue further prospective clinical trials evaluating HDAC inhibitors perhaps in combination with other agents, for the treatment of ACC. Such future trials should include additional tumor measurement criteria, such as volumetric tumor measurement and quality of life questionnaires, along with systematic molecular analysis and tumor sequencing.

## MATERIALS AND METHODS

### Eligibility criteria

Key eligibility criteria included: histologically or cytologically confirmed locally advanced, recurrent or metastatic ACC; age ≥ 18 years; measurable disease per RECIST v1.1; Eastern Cooperative Oncology Group (ECOG) performance status 0 to 2; life expectancy ≥ 12 weeks. Any number of prior chemotherapy regimens was allowed but not required. Laboratory parameters included adequate organ and marrow function defined as: leucocytes ≥ 3,000/μL, absolute neutrophil count ≥ 1,500/μL, platelets ≥ 100,000/μL, total bilirubin within institutional normal limits (WNL), liver enzymes ≤ 2.5X upper limit of normal, and creatinine WNL, or creatinine clearance ≥ 60 mL/min. Patients with previous brain metastases were eligible if they were treated and stable for ≥ 1 month with no requirement for steroids. All patients provided informed consent before treatment. The study complied with local institutional review board guidelines. Baseline formalin fixed paraffin embedded FFPE slides or blocks needed to be available for correlative studies.

### Study design and treatment

This was a multicenter, international, single-arm, phase 2 study. Patients received oral vorinostat 400 mg once a day continuously until disease progression, death, withdrawal of consent, or unacceptable adverse events. Each cycle was defined as 28 days. Imaging was obtained at baseline and repeated every 8 weeks for the first 6 cycles. After 6 months on study, imaging was extended to every 12 weeks. Study visits occurred every 2 weeks for the first 2 cycles, then every 4 weeks thereafter. Safety assessments included physical examinations, AE assessment, and laboratory measurements. AEs were graded according to the Common Terminology Criteria for Adverse Events (CTCAE) version 4.0. Vorinostat was distributed by Cancer Therapy Evaluation Program (CTEP) of the National Cancer Institute under a collaborative agreement with Merck & Co. Inc.

### Objectives and endpoints

The primary objective was to evaluate the efficacy as defined by response rate (RR) of vorinostat in patients with ACC. Secondary objectives were to evaluate time to tumor response, response duration (RD), stable disease duration, progression free survival, overall survival and to characterize safety and tolerability of vorinostat in ACC patients. For RD, SDD, and PFS, if progression had not occurred, then their duration was censored as of the date of the most recent tumor assessment. For patients still alive, their OS duration was censored as of the most recent date that the patients’ vital status had been confirmed.

### Statistical methods

#### Design

Complete + partial (CR+PR) rate was the primary statistical endpoint. We used a 2-stage Simon optimal design with p_0_ = 5%, p_1_ = 20%, alpha = 0.15, power = 0.90, and PET = 0.540. Stage 1 required 12 patients. At least 1 responder in Stage 1 would be needed to justify continuing to Stage 2, which would require 17 more patients. If < 3 responders, we would conclude that vorinostat has insufficient efficacy to justify further study.

#### Analysis

95% confidence interval (CI) estimates were calculated via Wilson's method. TTE endpoints were estimated using the Kaplan-Meier (K-M) method. Due to the small numbers of events, point estimates of survival statistics were estimated more conservatively using linear interpolation among successive event times on the K-M curves, and a slightly lower confidence level (90%) was used when determining CI’s. A waterfall plot was used to display the degree of response (percent change in tumor burden).

### Exploratory studies methods

#### Whole exome sequencing

The DNA from FFPE tumor tissue and from normal lymphocytes were extracted with QIAamp^®^ FFPE Tissue Kit and QIAamp^®^ DNA Blood Mini Kit respectively according to the supplier's instructions. All double strand DNAs were quantified using a QuBit^®^ fluorometer. In order to evaluate the clinical samples from the patients with differential responses to vorinostat WES of 5 available tumor normal pairs with sufficient tumor DNA (patients 001–002, 001–004, 001–006, 003–014, 003–027) from the FFPE tissue samples was done using Agilent_V5_PlusUTR_hs37d5_Baits. The sequencing was done through the TGen Collaborative Sequencing Center (for patients 001–004 and 001–006) and the Mayo Clinic Medical Genome Facility (MGF) at a mean depth of 60x coverage according to established protocols [52]. Observed variants were filtered based on a series of criteria including NextProt Feature Strength, Maximum Population Allele Frequency, and inclusion in the Catalogue of Somatic Mutations in Cancer (COSMIC) database [7]. The goal was to prioritize those variants that likely disrupt protein function, identify mutations targeting known cancer related genes and pathways, and filter out polymorphisms detected in patient matched normal samples and reported in dbSNP and other population based studies. Of significant interest were those somatic variants that introduced non-synonymous variants within well-annotated cancer associated genes.

#### Targeted resequencing

In addition to WES, we developed a 10 gene panel to validate mutations and to screen samples with sub optimal levels of DNA for this study. We selected 4 genes from our initial WES results for patients 001–004 and 001–006, and 6 additional genes from previously published ACC data for our 10 gene panel [5, 6]. Candidate driver aberrations targeting chromatin remodeling (*SMARCA2*, *KDM6A*, *CREBBP*), DNA repair and checkpoints (*TP53*, *MLF1*) and cell signaling (*PIK3CA*, *SH2D4A*, *FZD10*) were prioritized based on their biological functions and potential clinical utility. The final 10-gene ACC panel consisted of *SH2D4A, FZD10, TP53, PIK3CA, PTEN, SMARCA2, KDM6A, CREBBP, MLF1*, and *N4BP2*. Primers were designed for each of the 10 genes with coverage for all exons and untranslated sequencings (UTRs) using the Ion Ampliseq^TM^ Designer https://www.ampliseq.com/browse.action. Our targeted resequencing 10 gene FFPE specific input panel has 87.65% coverage with 644 amplicons ranging from 125–175 base pairs in length, for a total of 62.09 kb of sequence. This design provides validation of the 4 novel mutations identified in the initial whole exome data and increases the probability of identifying additional mutations that converge on each of the 10 genes in the panel. For exploratory purposes, patients were qualified as either “responders” (7/11 patients) or “non-responders” (4/11 patients) based on whether they had a PR by RECIST (*n* = 2), prolonged SD, clinical benefit to vorinostat or progressed quickly on study. To investigate the role of somatic mutations in responses to vorinostat we assembled archival FFPE blocks from 2 patients and FFPE slides from an additional 9 patients treated with vorinostat. We obtained patient matched normal blood samples as controls. In total we screened both tumor and normal tissues from each of 8 patients (from 6 “responders” and 2 “non-responders”), including the 5 cases that were analyzed by WES with this panel using the Ion Torrent platform. In the remaining 3 cases there was insufficient material for either WES or target resequencing. A full description of the methods can be found in the Supplementary File.

## SUPPLEMENTARY MATERIALS TABLE AND FIGURES


