# Supplementary material for: A phase 2 study of vorinostat in locally advanced, recurrent, or metastatic adenoid cystic carcinoma
Source: Oncotarget. 2017 Mar 22;8(20):32918–29. doi: 10.18632/oncotarget.16464 (PMC5464838; doi:10.18632/oncotarget.16464)
Supplement: Supplementary file 1 [file oncotarget-08-32918-s001.pdf]

A phase 2 study of vorinostat in locally advanced, recurrent, or metastatic adenoid cystic carcinoma

Supplementary Materials

Next generation sequencing (NGS) methods

NGS

Whole exomes. 200 ng of each DNA was used to construct exome libraries using Agilent SureSelect Human All Exon V5+UTR baits and XT adapters following the manufacturer's protocols. Final libraries were evaluated by Qubit, pooled, and evaluated on the Agilent TapeStation prior to clustering. Libraries with a 1% phiX spike-in were used to generate clusters on HiSeq Paired End v3 flowcells on the Illumina cBot using Illumina's TruSeq PE Cluster Kit v3. Clustered flowcells were sequenced by synthesis on the Illumina HiSeq 2000 using paired-end technology and Illumina's TruSeq SBS Kit, extending to 83 bp for each of two reads. FASTQ generation was performed using Illumina's BCLConverter tool and reads aligned with BWA using paired-gapped alignment [1]. Results were recalibrated using GATK [2, 3] and duplicates marked using Picard (http://broadinstitute.github.io/picard). For reads containing indels, microalignment was performed. Variants were detected using Seurat [4], MuTect [5], and Strelka [6] and annotated using Gencode version 3 by ENSEMBL and build 37.1. Variants detected by at least 2 of 3 callers are marked as PASS calls.

Targeted resequencing

We used established protocols for the Ion Torrent platform for all steps. Briefly, 50ng of each DNA was amplified with 2 separate pools of primers that were designed using the Ion Torrent Ion AmpliSeq™ Designer https://www.ampliseq.com/protected/startPage.action. After digestion barcodes were ligated to each library which were then washed and resuspended prior to quantification using TaqMan based assay supplied by the manufacturer. Sequencing templates were then prepared using the Ion OneTouch™ 2 instrument. Templates are recovered after amplification then either stored at 4C or enriched then sequenced using the Ion PGM™ chip and sequencing kit. The quality of the unenriched template is assessed by Qubit® 2.0 Fluorometer and the fluorophores Alexa Fluor® 488 and Alexa Fluor® 647. The ratio of the two different fluorophores yields the % templated. Templates with > 10% and < 30% efficiency are then used for sequencing. All custom sequencing runs are planned through the Ion Torrent server: http://172.16.36.146/plan/planned/. The BAM and BAM Index files for each sample were obtained through the server then uploaded into IGV 2.1 for final inspection.

REFERENCES

1. Li H, Durbin R. Fast and accurate short read alignment with Burrows-Wheeler transform. Bioinformatics. 2009; 25:1754–1760.
2. McKenna A, Hanna M, Banks E, Sivachenko A, Cibulskis K, Kernytsky A, Garimella K, Altshuler D, Gabriel S, Daly M, DePristo MA. The Genome Analysis Toolkit: a MapReduce framework for analyzing next-generation DNA sequencing data. Genome research. 2010; 20:1297–1303.
3. DePristo MA, Banks E, Poplin R, Garimella KV, Maguire JR, Hartl C, Philippakis AA, del Angel G, Rivas MA, Hanna M, McKenna A, Fennell TJ, Kernytsky AM, et al. A framework for variation discovery and genotyping using next-generation DNA sequencing data. Nature genetics. 2011; 43:491–498.
4. Christoforides A, Carpten JD, Weiss GJ, Demeure MJ, Von Hoff DD, Craig DW. Identification of somatic mutations in cancer through Bayesian-based analysis of sequenced genome pairs. BMC Genomics. 2013; 14:302.
5. Cibulskis K, Lawrence MS, Carter SL, Sivachenko A, Jaffe D, Sougnez C, Gabriel S, Meyerson M, Lander ES, Getz G. Sensitive detection of somatic point mutations in impure and heterogeneous cancer samples. Nature biotechnology. 2013; 31:213–219.
6. Saunders CT, Wong WS, Swamy S, Becq J, Murray LJ, Cheetham RK. Strelka: accurate somatic small-variant calling from sequenced tumor-normal sample pairs. Bioinformatics. 2012; 28:1811–1817.

Supplementary Table 1: Summary statistics of time to event (TTE) end-points

TTE End-point	*N*	Events	Point Estimate	90% Confidence Interval	
**SDD**	27	14			
**Median (months)**			11.4	7.1	20.5
**6 month rate**			75%	60%	91%
**12 month rate**			45%	25%	65%
**PFS**	30	16			
**Median (months)**			11.4	7.1	20.5
**6 month rate**			72%	58%	86%
**12 month rate**			46%	27%	64%
**OS**	30	3			
**Median (months)**			#	#	#
**6 month rate**			94%	86%	100%
**12 month rate**			88%	75%	100%

[#]Median not yet reached.

SDD- stable disease duration; PFS - progression free survival; OS - overall survival.

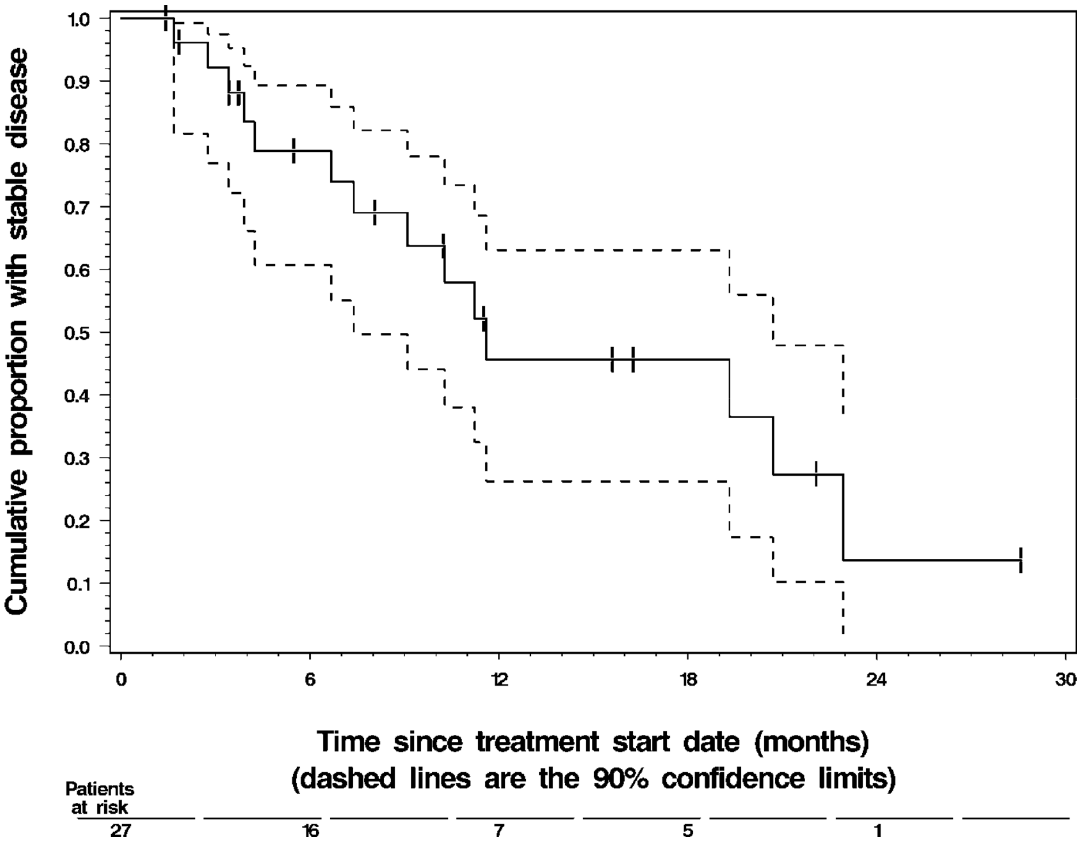

Supplementary Figure 1: Kaplan-Meier graph of stable disease duration (SDD) for all 27 patients whose best response was stable disease (SD). Dashed lines identify the pointwise 90% confidence limits for successive rates of freedom from progression.

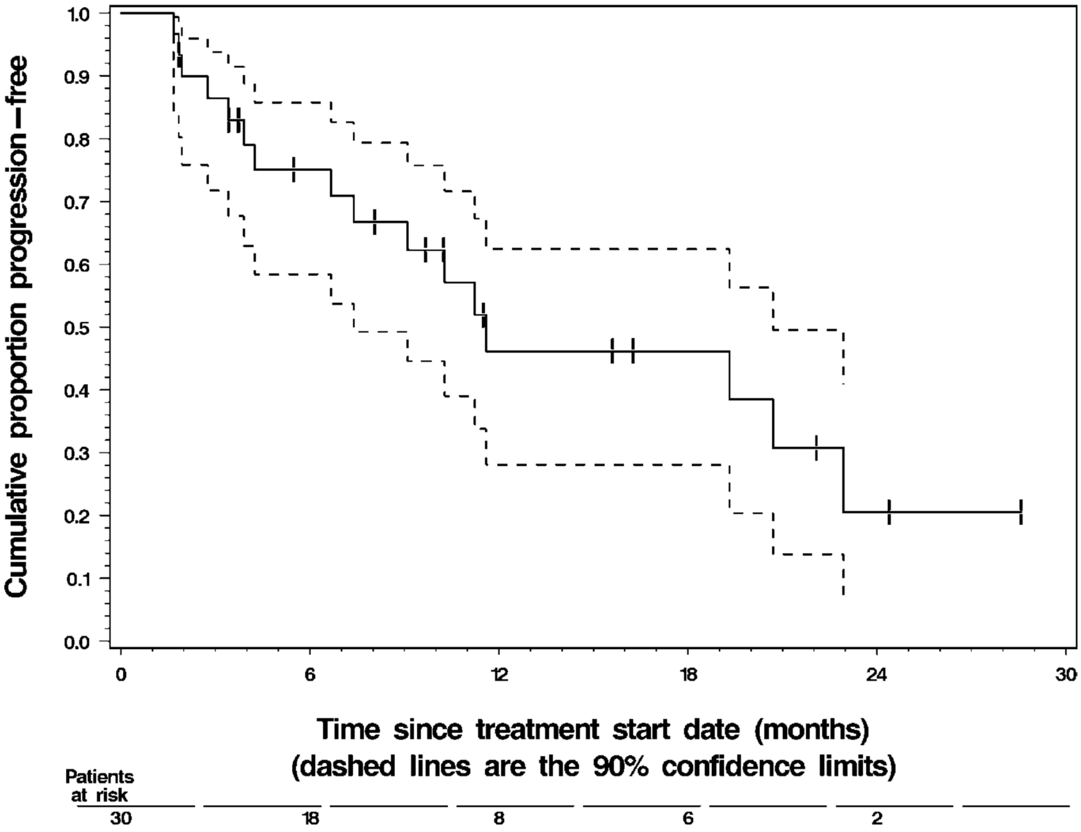

Supplementary Figure 2: Kaplan-Meier graph of progression-free survival (PFS) for all 30 treated patients. Dashed lines identify the pointwise 90% confidence limits for successive rates of freedom from progression.

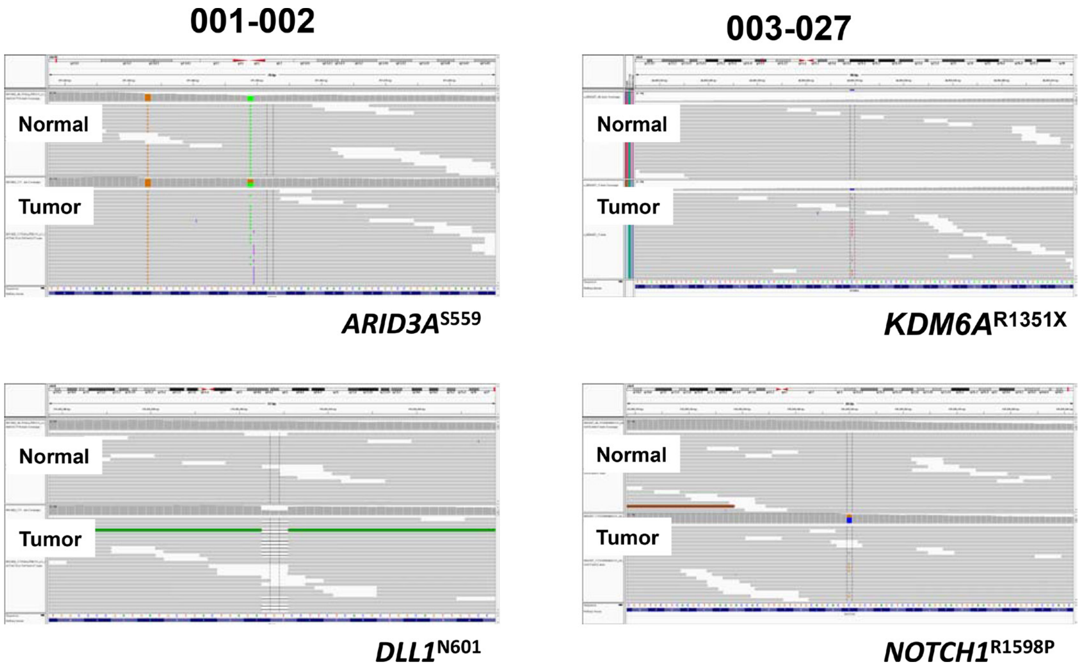

Supplementary Figure 3: Integrated Genomic Viewer (IGV) display of mutations detected in whole exome data. Mutations including indels and single nucleotide variants targeting NOTCH signaling and chromatin regulation in ACC responder (001-002) and non-responder (003-027) are shown.
